# Precise activity measurements of medical radionuclides using an ionization chamber: a case study with Terbium-161

**DOI:** 10.1186/s40658-022-00448-0

**Published:** 2022-03-14

**Authors:** Frédéric Juget, Zeynep Talip, Youcef Nedjadi, M. Teresa Durán, Pascal V. Grundler, Jan Rijn Zeevaart, Nicholas P. van der Meulen, Claude Bailat

**Affiliations:** 1 Institute of Radiation Physics, Lausanne, Switzerland; 2 Center for Radiopharmaceutical Sciences, ETH-PSI-USZ, Paul Scherrer Institute, Villigen-PSI, Switzerland; 3 Radiochemistry, South African Nuclear Energy Corporation (Necsa), Brits, South Africa; 4 Laboratory of Radiochemistry, Paul Scherrer Institute, Villigen-PSI, Switzerland

**Keywords:** ^161^Tb, Système International de Référence, Low-energy gamma, Activity measurement, Radionuclide dose calibrator

## Abstract

**Background:**

^161^Tb draws an increasing interest in nuclear medicine for therapeutic applications. More than 99% of the emitted gamma and X-rays of ^161^Tb have an energy below 100 keV. Consequently, precise activity measurement of ^161^Tb becomes inaccurate with radionuclide dose calibrators when using inappropriate containers or calibration factors to account for the attenuation of this low energy radiation. To evaluate the ionization chamber response, the sample activity must be well known. This can be performed using standards traceable to the Système International de Référence, which is briefly described as well as the method to standardize the radionuclides.

**Methods:**

In this study, the response of an ionization chamber using different container types and volumes was assessed using ^161^Tb. The containers were filled with a standardized activity solution of ^161^Tb and measured with a dedicated ionization chamber, providing an accurate response. The results were compared with standardized solutions of high-energy gamma-emitting radionuclides such as ^137^Cs, ^60^Co, ^133^Ba and ^57^Co.

**Results:**

For the glass vial type with an irregular glass thickness, the ^161^Tb measurements gave a deviation of 4.5% between two vials of the same type. The other glass vial types have a much more regular thickness and no discrepancy was observed in the response of the ionization chamber for these type of vials. Measurements with a plastic Eppendorf tube showed stable response, with greater sensitivity than the glass vials.

**Conclusion:**

Ionization chamber measurements for low-energy gamma emitters (< 100 keV), show deviation depending on the container type used. Therefore, a careful selection of the container type must be done for activity assessment of ^161^Tb using radionuclide dose calibrators. In conclusion, it was highlighted that appropriate calibration factors must be used for each container geometry when measuring ^161^Tb and, more generally, for low-energy gamma emitters.

## Introduction

Radionuclides are used extensively in nuclear medicine worldwide for diagnosis, using positron emission tomography (PET) or single-photon emission computed tomography (SPECT), and for therapy, using α-particles, β-particles or Auger electrons as locally-deposited energy to treat tumors and metastases using the locally deposited energy. The need for precise quantification of the radioactivity injected into the patient is becoming more important to optimize the image quality in diagnosis and to perform personalized therapy and accurate dosimetry.

In radionuclide production centers, preclinical research laboratories and nuclear medicine facilities, the activity of radionuclides is measured using radionuclide dose calibrators (RDCs). It is important to stress that such an instrument has to be calibrated with well-known samples in a similar physical or chemical form as used in the laboratory in question, using the same container type. Measurements with RDCs are sensitive to geometrical variations, such as the position of the container in the chamber well, the container type, syringe or vial and their shape, wall thickness, material components and container-filling volume. Most RDCs are usually calibrated with one calibration factor per radionuclide, usually in a "vial geometry" provided by the manufacturer. However, the container used for activity measurement has to be well selected, particularly for the measurement of low-energy gamma, X-ray and β-emitters, for which the attenuation will depend on the container type.

Several studies were carried out using different RDC types, with different radionuclides and geometries. Santos et al. reported the effects of geometry for ^99m^Tc (main gamma emission is at 141 keV) [1]. This study showed that the volume of the solution must be placed in an area sufficiently deep in the well of the RDC chamber in question. Thus, if the syringe holder is too high or the syringe too full, large measurement errors of about 10% or more can occur. Cessna et al. [2] reported the calculation of a calibration factor for plastic syringes with ^18^F and compared it with the manufacturer's value given for a glass vial. The value obtained was approximately 12% higher for the two tested instruments, which meant that the response of the RDC was greater than that expected for the manufacturer's calibration factor. This is due to the fact that the manufacturer calibration factor value was obtain with a glass vial while, in the case of a syringe, the radiation is less attenuated. Moreover, the solution was not located in the same position in the RDC well as for the glass vial. The comparison of ^111^In activity measurements in a RDC with glass vials and plastic syringes showed that, for low-energy gamma-rays (23–26 keV of ^111^In), the measurement error can reach 35% even when using glass vials [3]. Olsovcova highlighted that the effect of low-energy gamma-rays of ^123^I (~ 30 keV) for plastic syringes can lead to an activity measurement error of 40% [4]. The effect of low energies (20–40 keV) for different container types were also discussed in [5]. An error of up to 25% for syringes with ^111^In was observed, confirming the results from [3, 4] described previously. A recent article [6] reported the activity measurements of ^99m^Tc, ^111^In, ^18^F, and ^68^Ga with different syringes and vials using RDCs, showing discrepancies of up to 30% for ^111^In for syringes, due to the fact that only one calibration factor was available from the manufacturer and was calculated for glass vials. Significant differences were also reported for the other radionuclides depending on the geometries used. Vargas et al. performed a similar study to investigate the accuracy of RDCs for ^99^Tc, ^111^I, ^123^I, ^124^I, ^131^I ^177^Lu and ^90^Y [7]. An international multi-center investigation was reported using 32 RDCs from 8 hospitals located in The Netherlands, Belgium and Germany, respectively. It was concluded that, for ^111^I, ^123^I, ^124^I and ^90^Y, the response of RDCs was particularly sensitive to the sample and detector geometry.

This brief introduction to RDC measurements highlights the important requirements for the accurate use of these devices. For measurements of almost pure γ-sources like ^57^Co, ^60^Co, ^99m^Tc or ^131^I, where electron emission energy is below 500 keV, the value of the calibration factors given by the manufacturer can generally be used for syringes and vials. On the other hand, for low-energy gamma emitters (below 100 keV) large deviations can be measured (up to 40%). In order to calibrate the RDC for each geometry, a well-known radioactive solution is needed. Such a standard, usually provided by Nuclear Metrology Institutes (NMIs), should be traceable to international comparisons. In practice, standards are provided by NMIs or Designated Institutes by the NMI in each country. To ensure good measurements practices by NMIs, the governments and the regulatory organizations worldwide have defined a comparison program at the General Conference on Weights and Measures (CGPM) to ensure the traceability of measurement standards. The Bureau of Weights and Measures (BIPM) produced a dedicated document, International Committee for Weights and Measures Mutual Recognition Agreement (CIPM MRA), initially written in 1999, revised in 2006 and signed by 106 institutes from 62 member states, 40 associated states of the CGPM and 4 international organizations [8]. The CIPM MRA is the framework through which NMIs demonstrate the international equivalence of their measurement standards and the calibration and measurement certificates. The outcomes of the arrangement are internationally recognized (peer-reviewed and approved).

Calibration and Measurement Capabilities (CMCs) describe the radionuclide measurement capabilities of the NMIs and then the realization of standards. This capability is evaluated with the measurement comparisons between NMIs. These results provide average values to validate the different standardization measurement techniques. The CIPM MRA established a program by which the results from one radionuclide metrology laboratory can be considered in the same context as a measurement of the same quantity at another NMI.

When a NMI standardizes a radionuclide and wants to participate in a comparison study to obtain the CMC, it submits a sample of its standardized solution to the Système International de Référence (SIR). The SIR is the measurement system, based at BIPM, where the comparison measurement is performed with a dedicated ionization chamber and the comparison results are evaluated. Since the CIPM MRA was signed in 1999, the SIR has been designated as the method by which NMIs compare their results [9, 10]. Approved CMCs and supporting technical data are publicly available from the CIPM MRA database, the Key Comparison Data Base (KCDB) [11]. In practice, when a NMI standardizes a radionuclide with primary measurement techniques, an aliquot of the standardized solution is used to fill a dedicated ampoule with a specific geometry and a precisely-known mass. The sealed ampoule is sent to the BIPM for measurement with the SIR. The same standardized solution is used to fill dedicated ampoules at the NMI, which are measured in a dedicated ionization chamber.

**Fig. 1:**
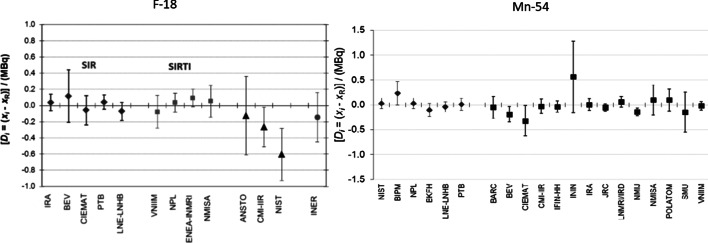
International comparison for ^18^F and ^54^Mn for IRA and other NMIs in the Système International de Référence. The vertical axis reports the difference between the reference activity (*X*_R_) and the measured activity (*X*_i_)

At the Institute of Radiation Physics (IRA; Lausanne, Switzerland) this dedicated ionization chamber is called Chambre d’Ionisation de Référence (CIR). It has been in operation since 1983 and is regularly maintained and checked to ensure its stability [12]. Using the known activity of the ampoules, a calibration factor, called equivalent activity (*A*_e_), is calculated with an accuracy of around 0.5%. Once *A*_e_ is known, the CIR is used to calculate the unknown activity of any solution containing the radionuclide of interest. This chamber can therefore, be used to measure the activity of solutions and to produce standards that are traceable to the SIR. In Switzerland, IRA is the designated institute, by the Swiss Federal Office of Metrology (METAS), for radioactivity measurement. IRA has more than 20 CMCs and provides standards for the calibration of radioactivity measurement instruments. Figure 1 shows two examples of comparison measurements performed for ^18^F and ^54^Mn by IRA and other NMIs [13, 14]. As the radioactivity users have to establish a quality assurance system for activity measurement, the measurement devices must be calibrated using reference standards and also be regularly verified as defined in the Swiss regulation [15, 16]. According to this regulation, the activity of each dose unit must be determined before medical use. The measurement can be made by an instrument calibrated with nationally-recognized standards or manufacturer’s instructions. RDC is the device recognized by the authority to assay activity before administration to patients. The regulation in Switzerland allows a tolerance of ± 10% [15, 16] for the activity measured with a RDC, while the International Atomic Energy Agency (IAEA) recommends ± 5% [17]. Clearly, the use of quality standards for the calibration of RDCs is required. Providing certified standards ensures the accurate calibration of radioactivity measurement devices in the scientific community, allowing quality assurance for the end users, such as nuclear medicine services.

The beta-emitting ^161^Tb (*E*_βav_^−^: 154 keV (100%), *T*_1/2_ = 6.953 d [18]) is an attractive radionuclide for targeted radionuclide therapy. It has been considered as an alternative to the clinically-approved ^177^Lu [19]. Dose calculations have demonstrated that ^161^Tb can be more effective than ^177^Lu for small tumor lesions due to the emission of low-energy conversion and Auger electrons [20, 21]. Preclinical in-vivo and in-vitro studies performed using [^161^Tb]Tb-PSMA-617 have also shown very promising results compared to [^177^Lu]Lu-PSMA-617 [22, 23]. Moreover, ^161^Tb can be used for monitoring the activity distribution and dosimetry thanks to the emission of gamma-radiation. Marin et al. established a SPECT/CT protocol for imaging of ^161^Tb using an energy window centered at 74.6 keV [24]. Recently, the first-in-human application of [^161^Tb]Tb-DOTATOC demonstrated the ^161^Tb imaging properties using SPECT/CT [25]. The results showed high quality images, and it was even possible to visualize small metastases in the liver and bones. Moreover, the development of a protocol towards Good Manufacturing Practice (GMP)-compliant production of [^161^Tb]-DOTATOC is in progress [26].

As a result, at this stage of ^161^Tb research, the precision of ^161^Tb activity measurements is crucial to shorten the transition period of this potential therapeutic radionuclide to the clinics. More than 99% of the emitted gamma and X-rays of ^161^Tb have an energy below 100 keV [27, 28] (Table 1). An adequate container has to be used for the activity measurement using dose calibrators, as large attenuation could occur depending on the container characteristics. As a result, this study aims at evaluating the effect of sample container and source geometry on the RDC measurements of ^161^Tb.

**Table 1:** Gamma-ray and X-ray intensities for ^161^Tb (only the main gamma-emission lines are shown)

Energy (keV) [26]	Emission intensities (%) [27]	s rel. (%)
48.91533	17.73	2.1
45.999	12.779	2.1
74.56669	10.28	1.7
45.2083	7.747	2.2
52.191	3.741	2.0
57.56669	2.066	1.9
53.6353	1.0185	1.9
59.243	0.2002	1.8
87.941	0.1996	1.6
103.065	0.1077	1.5

**Fig. 2:**
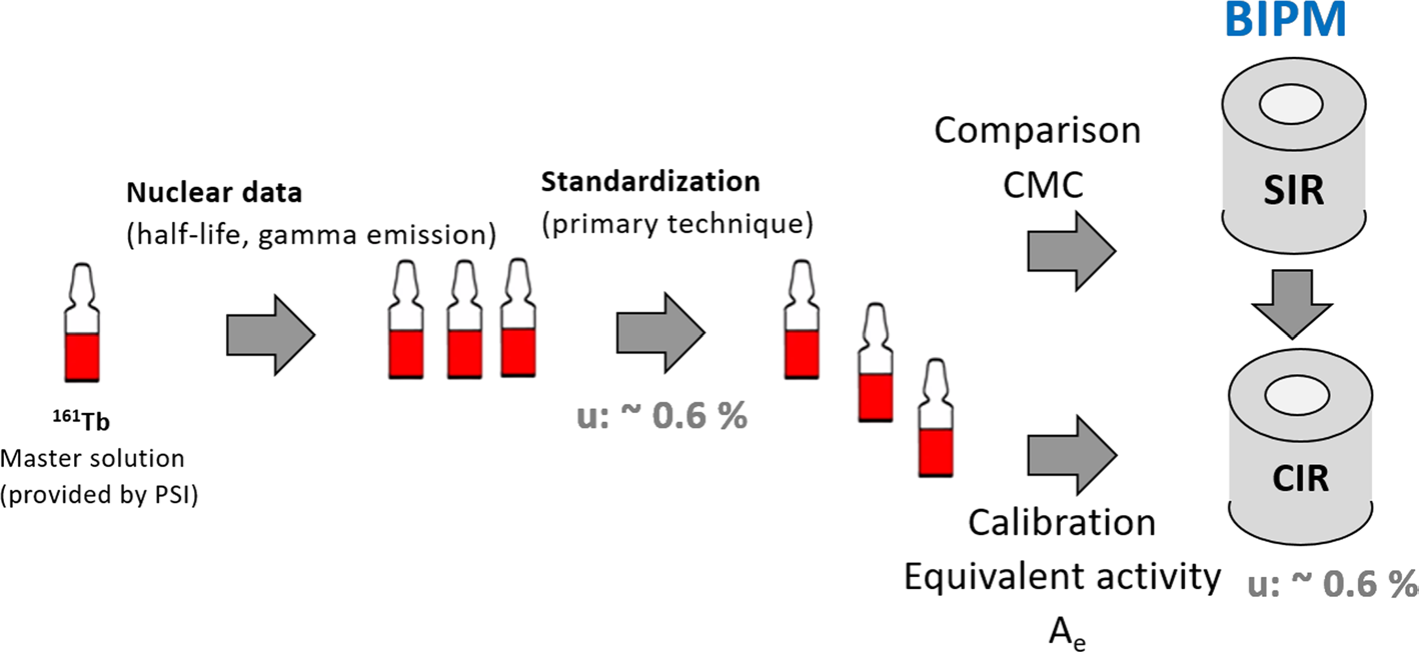
Scheme of the realization of a ^161^Tb standard (*CMC* Calibration and Measurement Capabilities, *BIMP* The Bureau of Weights and Measures, *SIR* Système International de Référence, *CIR* Chambre d’Ionisation de Référence)

Precise nuclear data, such as half-life and decay emissions, play a major role towards the accuracy of the activity measurement. Recently-performed half-life, emission intensities measurements and standardization of ^161^Tb at IRA [18, 28, 29] enabled us to submit a ^161^Tb sample to the SIR as well as to calculate an *A*_e_ for the CIR, which is briefly summarized in the following section. Thus, IRA has the ability to produce ^161^Tb standards for instrument calibration (Fig. 2). Several standardized ^161^Tb solutions were used to study the response of an ionization chamber for different container types to pin down the attenuation effect for the low-energy gamma emissions, showing the necessity to have a dedicated factor for each container.

### A brief review of ^161^Tb precise activity measurement

#### Half-life determination

To realize a standard of ^161^Tb, its half-life was first measured at IRA using a chemically and radionuclidically purified sample provided by the Paul Scherrer Institute (PSI) [18]. Terbium‐161 was produced using the ^160^Gd(n,γ)^161^Gd → ^161^Tb nuclear reaction by neutron irradiation of enriched ^160^Gd targets (98.2%, Isoflex, San Francisco, CA, USA) at the SAFARI‐1 reactor (Necsa, Pelindaba, South Africa). After a chemical separation process, only ^160^Tb (*T*_1/2_ = 72.3 h) was identified as a radionuclidic impurity using High Purity Germanium (HPGe) detector with an ^160^Tb/^161^Tb activity ratio of 4.93(15).10^–5^. Its contribution was taken into account in the ^161^Tb half-life calculation. Three different independent systems (CIR, portable ionization chamber (TCIR) and a CeBr_3_ γ-emission detector with digital electronics) were used for the measurements performed over a period of more than two times the ^161^Tb half-life, giving an improved result with low uncertainty (0.028%) [18].

#### Emission intensity measurements

A total of 28 gamma-rays and 4 X-rays were measured using an HPGe detector and compared with previous ^161^Tb emission intensity measurements [28]. A large reduction of the uncertainties was obtained thanks to the high radionuclidic purity of the source (^160^Tb ≤ 0.007%), a highly precise half-life value of ^161^Tb [18], a rigorous Monte Carlo calculation for the coincidence summing correction and an accurate precise activity measurement [29].

#### Activity standardization

After the half-life determination, a ^161^Tb sample was standardized at IRA using the β–γ coincidence technique with analogue and digital acquisition systems, as well as with the Triple-to-Double Coincidence Ratio method (TDCR) [29]. The sample solution containing a ^160^Tb impurity with a ^160^Tb/^161^Tb activity ratio of 4.53(20).10^−5^ was also provided by PSI. Using the standardized solution, an equivalent activity value was calculated for the CIR chamber.

#### Precise activity measurement of ^161^Tb

The ^161^Tb half-life was measured as 6.953(2) days, corresponding to a relative uncertainty of 0.03% [18]. The coincidence measurements with analogue electronics and the TDCR method showed a good consistency and were compatible with the digital coincidence results within uncertainties. The final result gave an activity measurement with an uncertainty of 0.58%. A sample of this standardized solution was submitted to the BIPM to be measured by the SIR.

Two ampoules filled with this solution were measured in the CIR chamber in order to determine the equivalent activity for ^161^Tb (i.e. its calibration factor). Both gave consistent results and the obtained equivalent activity value is *A*_e_ = 219.05 ± 1.32 (0.602%) MBq [29]. These results give IRA the capability to produce ^161^Tb standards with a precision of 0.6%.

## Materials and methods

### ^161^Tb activity measurement with ionization chamber using different vials

**Fig. 3:**
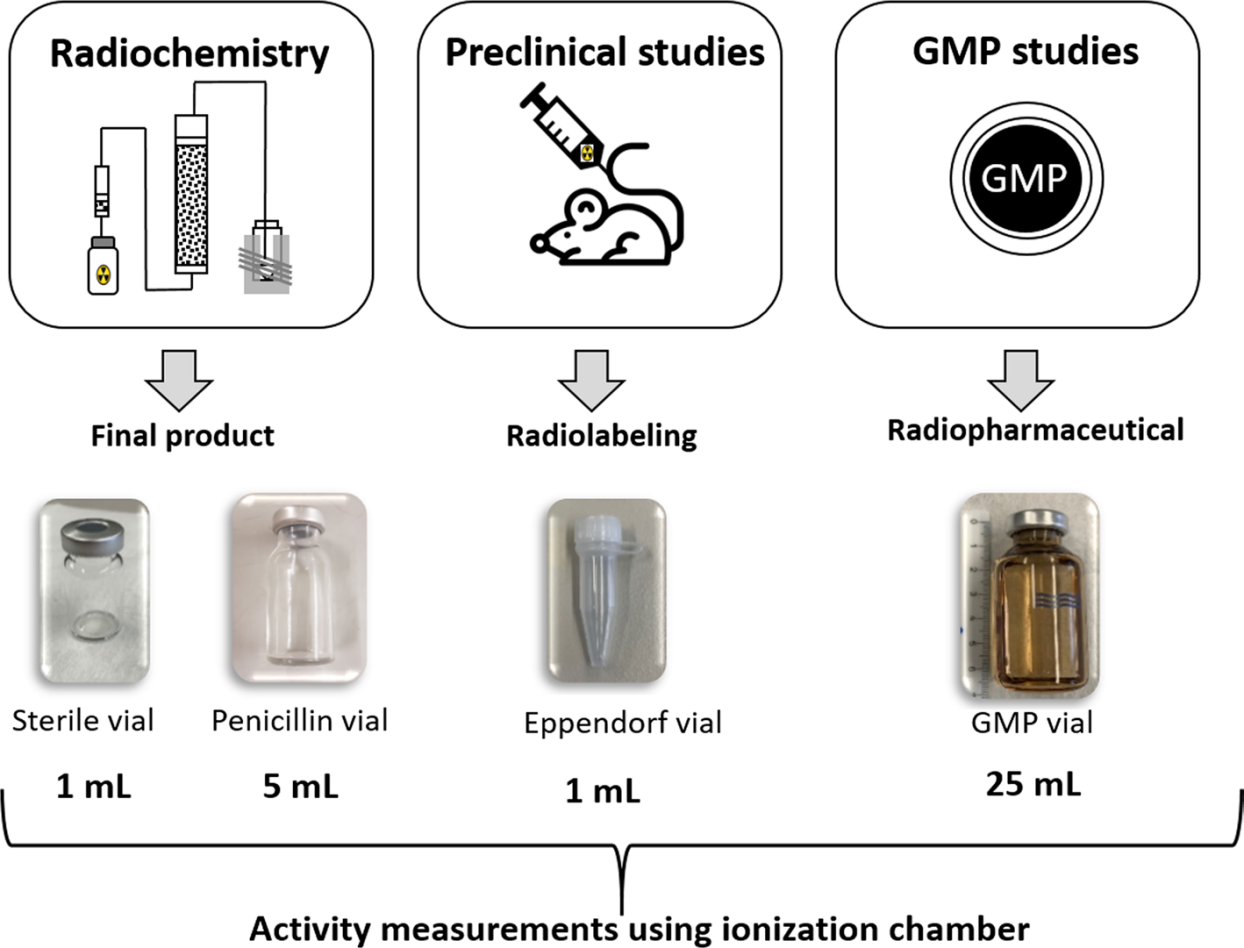
Scheme of the different container types used to assess the activity at different stages of ^161^Tb research

In this study, four different vials, used for the different stages of ^161^Tb research such as radiochemistry (radiochemical separation), preclinical studies and GMP studies (production) (Fig. 3), were chosen for the activity measurement of ^161^Tb using an ionization chamber whose characteristics are described in [30, 31].

#### Activity measurement in penicillin vials

During the standardization process at IRA [29], a solution of ^161^Tb consisting of 0.1 mol L^−1^ HCl solvent with a Tb^3+^-ion concentration of 25 μg g^−1^ was measured using different geometries. The density of the solution was 1.000(6) g cm^−3^. Two 10 mL penicillin glass vials, provided from Flaigg AG (Aesch, Switzerland) [32], with a diameter of 25.2 mm and a height of 52.8 mm, were filled with 5 g of the solution and their activity measured in the portable ionization chamber TCIR, which was able to accurately measure the current produced in the gas chamber [30, 31]. The mass of the solution deposited in each vial was accurately weighed in order to normalize the measured current and compare the two measurements.

#### Characterization of the penicillin vial

**Fig. 4:**
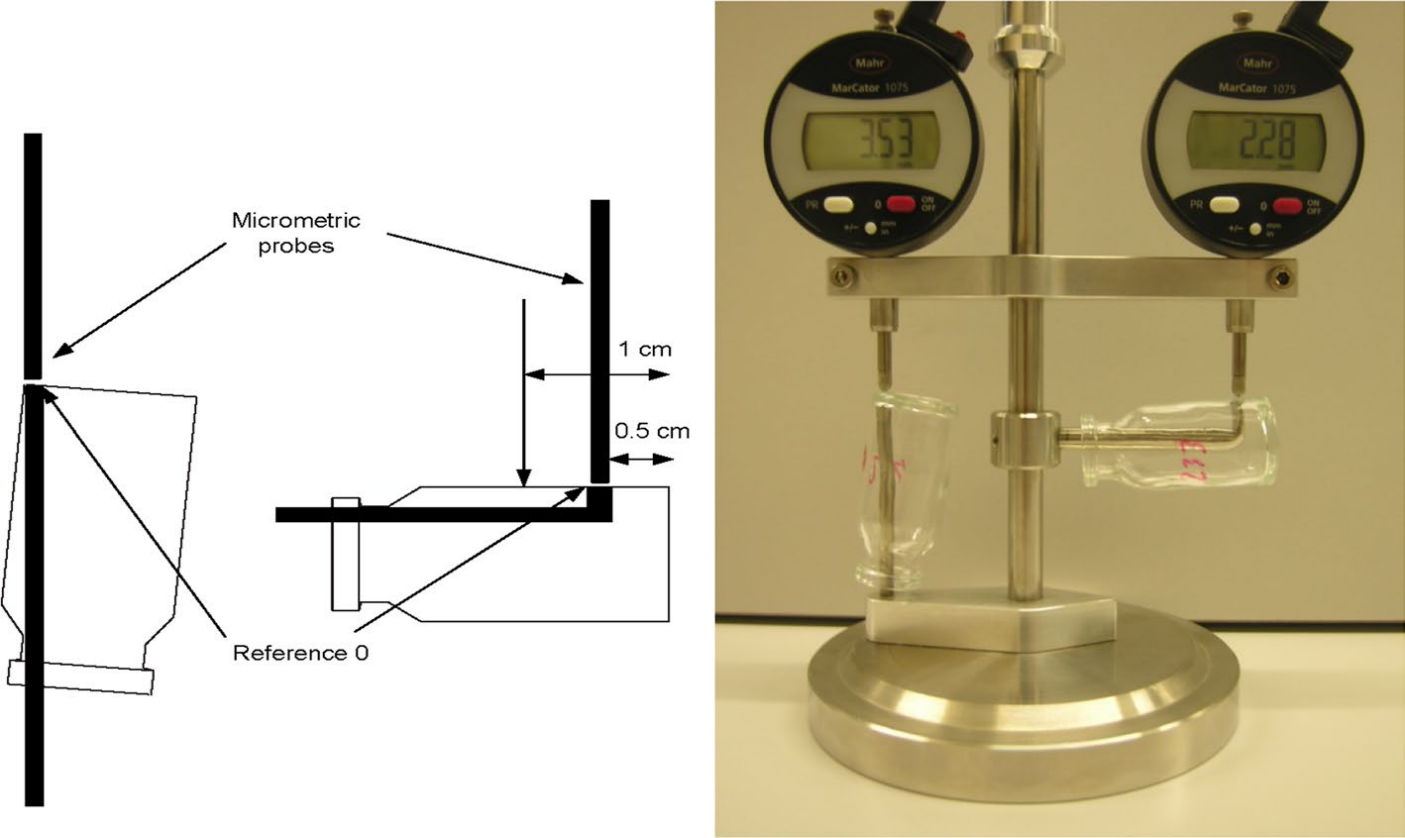
Left: scheme showing the locations of the probe for measurements at 0.5 and 1 cm with respect to the bottom of the vial and location of the probe for the bottom thickness measurement. For each position the vial is rotated manually to perform 10 measurements along its circumference. Right: picture of the measurement tool

The penicillin vials were characterized by measuring their wall thickness with a dedicated tool made at IRA (Fig. 4). Ten points were measured over the circumference at two positions, 0.5 cm and 1 cm from the bottom. Ten other points over the circumference of the bottom thickness were also measured to check its regularity and to select the vials with an uniform thickness.

#### Activity measurement in a sterile glass vial

A 5-mL glass vial, obtained from Infochroma AG (Goldau, Switzerland) [33], with an external diameter of 20 mm and a height of 38 mm was used in this work and is referred to as a sterile glass vial. The regularity of this vial was also characterized by measuring its wall thickness, as performed with the penicillin vial.

A ^161^Tb solution (1 g) was used to fill six sterile glass vials to assess their suitability for low-energy emission measurement. The solution consisted of 0.1 mol L^−1^ HCl solvent with a Tb^3+^-ion concentration of 25 µg g^−1^ with an activity concentration of 24.387 ± 0.146 (0.60%) MBq/g at the reference date. The concentration was measured at IRA with the CIR chamber, using the ^161^Tb equivalent activity as explained in the previous section. All the vials were weighed before and after filling to determine the precise mass of their content. Each vial was measured four times with the TCIR at different dates to check the consistency of the measurements over time.

#### Activity measurement in Eppendorf vial

**Fig. 5:**
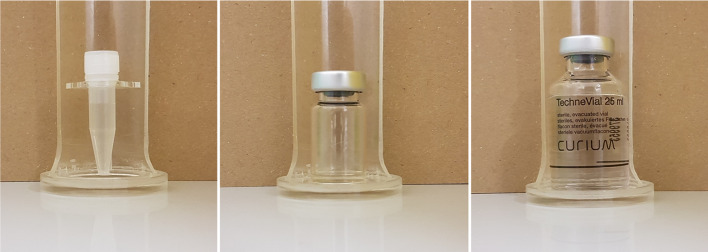
Eppendorf tube (left), sterile glass vial (middle) and GMP vial (right), in their respective holders before insertion inside the well of the ionization chamber

To show the effect of the container geometry on low-energy gamma emissions with a lighter container, two plastic Eppendorf tubes were filled with 1 g of the same solution as the sterile glass vials to measure the difference in the response of the ionization chamber. The Eppendorf is a tube of 43.5 mm in height and 10 mm external diameter with a conical end. A dedicated holder was designed to place the Eppendorf tube at a similar position to the one of the sterile glass vial inside the well of the TCIR ionization chamber (Fig. 5).

#### Activity measurement in a GMP vial

The containers called GMP vials (evacuated vials 25 mL (E6-12701)) were provided by Curium/b.e. imaging AG (Schwyz, Switzerland). They are used for the pharmaceutical production before injection into the patient. Usually, this vial is filled with 20 mL solution. The GMP vial has a diameter of 32.5 mm a height of 57 mm. The regularity of this vial was also checked by measuring its wall thickness, as done for the penicillin and the sterile glass vials.

A ^161^Tb solution with the same chemical composition as the one used for the sterile glass vials was used to fill the four GMP vials at 20 mL. The activity concentration was measured with the CIR and was determined to be 7.307 ± 0.044 (0.60%) MBq/g at the reference date. Each vial was weighed before and after filling to determine the precise mass of its content. The GMP vial was inserted into the TCIR chamber using the same holder as used for the other vials (Fig. 5).

## Results

### ^161^Tb activity measurement with ionization chamber using different vials

#### ^161^Tb activity measurement in penicillin vial

Table 2 gives the normalized current at the same reference date, obtained for several measurements of two penicillin vials, showing a discrepancy of 4.5% between the two vials.

**Table 2:** Normalized current produced in the ionization chamber for two penicillin vials filled with the same solution of ^161^Tb

Vial #	Meas. date	Current (pA/MBq)	Uncertainty	Rel. uncertainty	Average (pA/MBq)	Stand. dev.
**1**	23.08.2019	0.912	0.001	0.08%	0.911	0.001
**1**	24.08.2019	0.910	0.001	0.08%		
**1**	28.08.2019	0.910	0.001	0.08%		
**2**	23.08.2019	0.869	0.001	0.08%	0.869	0.001
**2**	24.08.2019	0.869	0.001	0.08%		
**2**	28.08.2019	0.869	0.001	0.08%		
				Diff:	4.5%	

#### Characterization of the penicillin vials

**Fig. 6:**
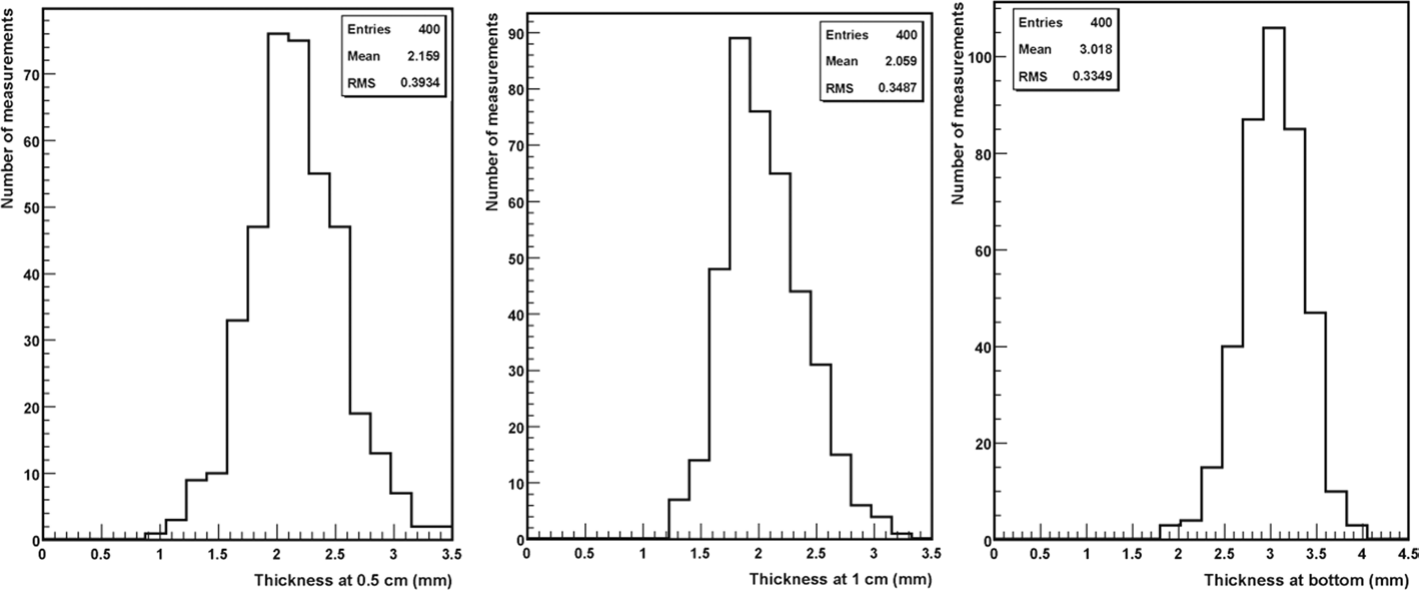
Distributions of the thicknesses measured for the three positions of the probes for 40 vials

Figure 6 shows the distribution of the values obtained for 40 vials for the three measurements considered, namely, thickness at 0.5 cm, thickness at 1 cm and bottom thickness (Fig. 4). The Root Mean Square (RMS) of the distributions (> 0.3 mm) shows the large spread of the distributions. However, these distributions are not useful to see how thickness can vary within the same vial. Therefore, the following quantities are defined to characterize the fluctuations in one vial:

$$\begin{aligned} P05_{i} & = {1}0\;{\text{measurements}}\;{\text{at}}\;0.{5}\;{\text{cm}}\;\left( {i = 1, \ldots 10} \right) \\ M05 & = {\text{average}}\;{\text{of}}\;{\text{the}}\;{1}0\;{\text{measurements}}\;{\text{at}}\;0.{5}\;{\text{cm}} \\ P10_{i} & = {1}0\;{\text{measurements}}\;{\text{at}}\;{1}\;{\text{cm}}\;\left( {i = 1, \ldots 10} \right) \\ M10 & = {\text{average}}\;{\text{of}}\;{\text{the}}\;{1}0\;{\text{measurements}}\;{\text{at}}\;{1}\;{\text{cm}} \\ F10_{i} & = {1}0\;{\text{measurements}}\;{\text{of}}\;{\text{the}}\;{\text{bottom}}\;\left( {i = 1, \ldots 10} \right) \\ MF & = {\text{average}}\;{\text{of}}\;{\text{the}}\;{1}0\;{\text{measurements}}\;{\text{of}}\;{\text{the}}\;{\text{bottom}} \\ C_{1} & = \mathop \sum \limits_{i = 1}^{10} \frac{{\left| {M05 - P05_{i} } \right|}}{10} = {\text{average}}\;{\text{of}}\;{\text{the}}\;{\text{difference}}\;{\text{of}}\;P05_{i} \;{\text{with}}\;{\text{respect}}\;{\text{to}}\;{\text{the}}\;{\text{average}}\;M05 \\ C_{2} & = \mathop \sum \limits_{i = 1}^{10} \frac{{\left| {M10 - P10_{i} } \right|}}{10} = {\text{average}}\;{\text{of}}\;{\text{the}}\;{\text{difference}}\;{\text{of}}\;P10_{i} \;{\text{with}}\;{\text{respect}}\;{\text{to}}\;{\text{the}}\;{\text{average}}\;M10 \\ C_{3} & = \mathop \sum \limits_{i = 1}^{10} \frac{{\left| {MF - F10_{i} } \right|}}{10} = {\text{average}}\;{\text{of}}\;{\text{the}}\;{\text{difference}}\;{\text{of}}\;F10_{i} \;{\text{with}}\;{\text{respect}}\;{\text{to}}\;{\text{the}}\;{\text{average}}\;MF \\ E_{1 } & = {\text{Standard}}\;{\text{deviation}}\;{\text{of}}\;\left| {M05 - P05_{i} } \right| \\ E_{2} & = {\text{Standard}}\;{\text{deviation}}\;{\text{of}}\;\left| {M10 - P10_{i} } \right| \\ E_{3 } & = {\text{Standard}}\;{\text{deviation}}\;{\text{of}}\;\left| {MF - F10_{i} } \right| \\ \end{aligned}$$

It was observed that, for some vials, the difference of the thickness was greater than 1 mm between two positions on the circumference. The bottom thickness was more regular, as the average difference and the standard deviation were 0.125 mm and 0.08 mm, respectively, compared with 0.27 mm and 0.16 mm for the side wall. These measurements showed that very few vials have regular thickness. In order to select regular vials, the following 6 criteria were used:

$$\begin{aligned} C_{1} & < 0.{2}\;{\text{mm}}\quad E_{1 } < 0.{1}\;{\text{mm}} \\ C_{2} & < 0.{2}\;{\text{mm}} \quad E_{2} < 0.{1}\;{\text{mm}} \\ C_{3} & < 0.{15}\;{\text{mm}}\quad E_{3 } < 0.0{75}\;{\text{mm}} \\ \end{aligned}$$

**Fig. 7:**
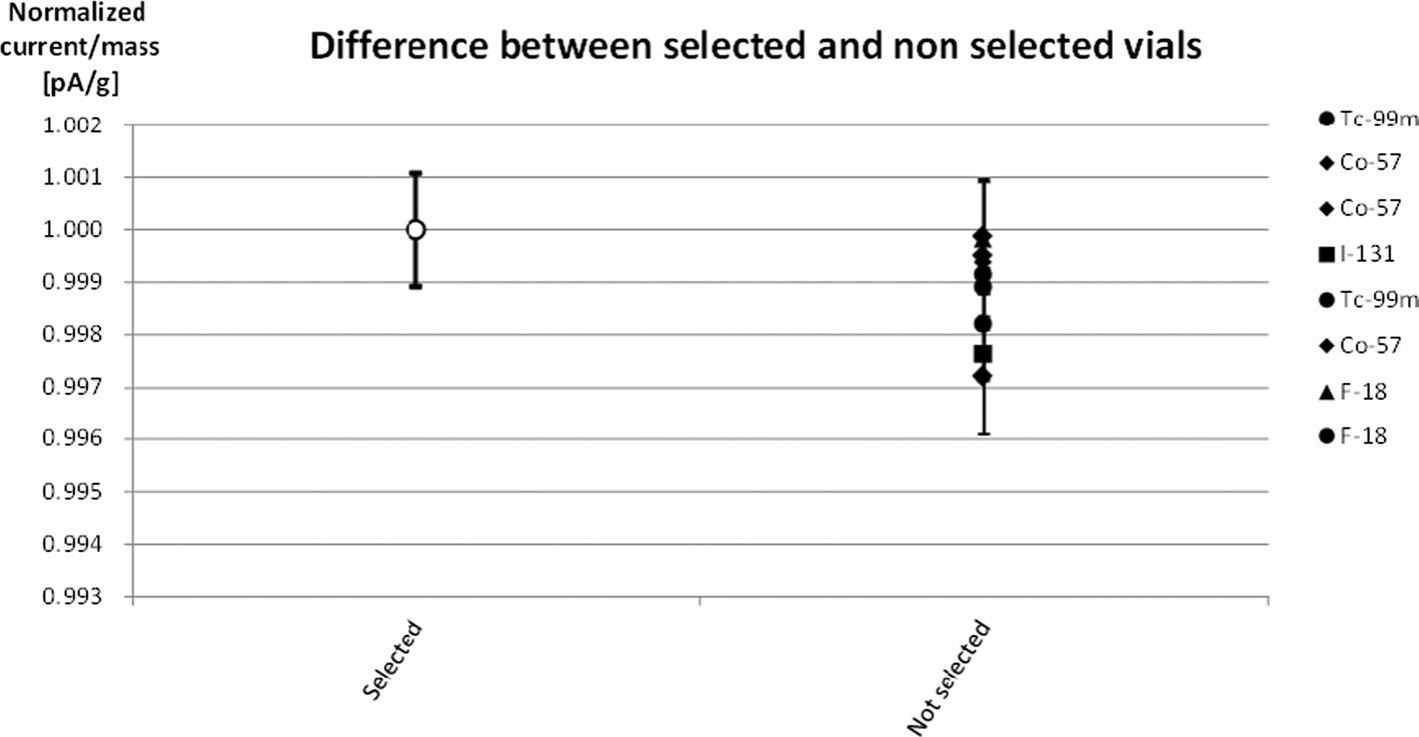
Ratio between the normalized current produced in the ionization chamber by selected and non-selected vials for different radionuclides

Fewer than 9% of the measured vials, over a sample of 223 vials, fulfilled these criteria. These selection criteria were validated using ^99m^Tc, ^57^Co, ^18^F and ^131^I. Selected and non-selected vials were filled with the same solution and measured in the ionization chamber. The results show differences between those vials satisfying the criteria and those not, but they are still less than 0.5% (Fig. 7). As this test was performed with radionuclides having gamma emissions above 100 keV, we used a ^161^Tb solution to ascertain whether low-energy gamma emissions could be measured with this vial type.

The results show that it is not appropriate to use penicillin vials for ^161^Tb, since they satisfy the geometry selection criteria but give a difference of 4.5% in the response current. It was, therefore, decided to use a more regular vial, as presented in the next section.

#### ^161^Tb activity measurement in sterile glass vials

**Fig. 8:**
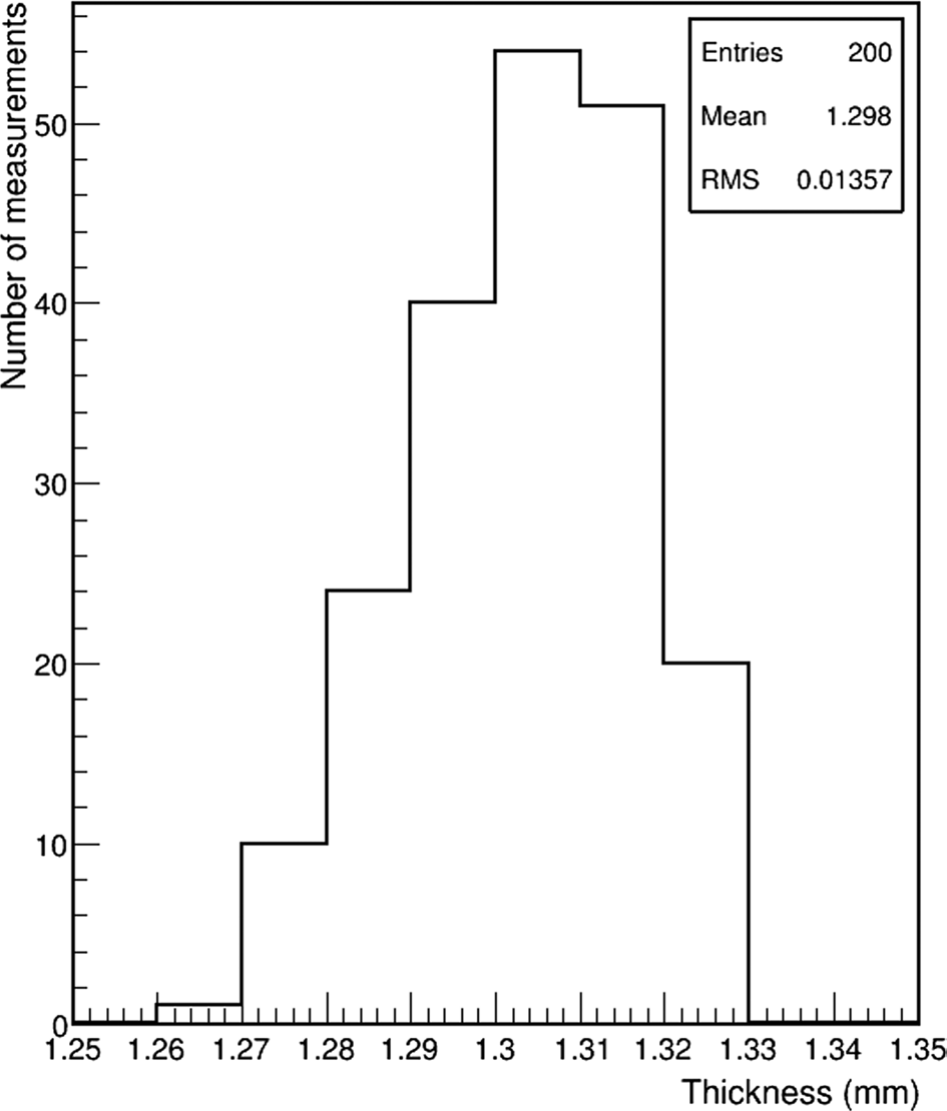
Distribution of the thickness measured for 10 points over the circumference of 20 sterile glass vials

The thickness of 20 sterile glass vials was measured to check their regularity. The results are shown in Fig. 8. The measurement of 10 points over the circumference showed very good regularity, the RMS of the distribution was less than 14 μm. The thickness was also thinner than for the penicillin vials: 1.3 mm compared to 2 mm. For all the 20 measured vials, the maximum fluctuation of the thickness was less than 50 μm. These vials were much more consistent than the penicillin ones and also have a thinner wall, which would attenuate the low –energy gamma rays less and produce more signal in the ionization chamber.

**Fig. 9:**
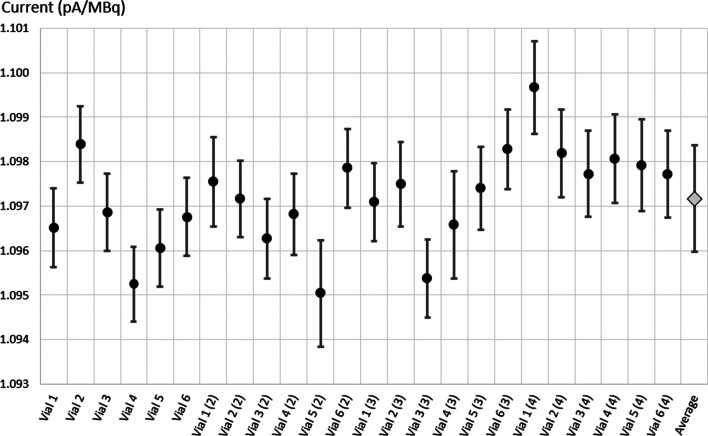
Normalized current values for 6 sterile glass vials filled with ^161^Tb. Each vial was measured 4 times at regular intervals

Figure 9 shows the results obtained using six sterile glass vials. The maximum deviation between the 24 measurements was 0.4%.

#### ^161^Tb activity measurement in the Eppendorf tube

**Fig. 10:**
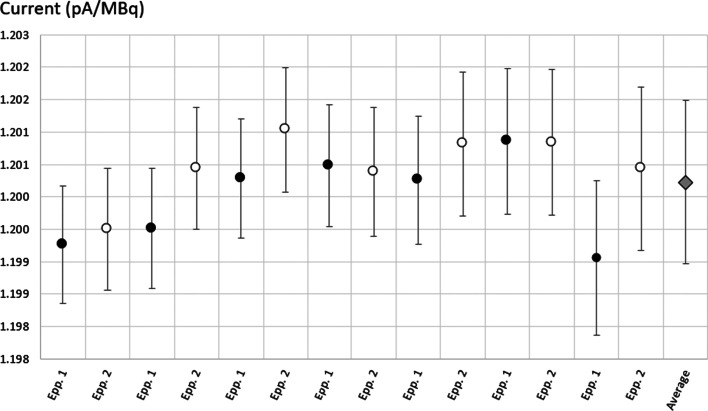
Normalized current values for two Eppendorf tubes filled with ^161^Tb. Each point represents seven measurements of both Eppendorf tubes performed at regular intervals over a period of 2 weeks

As shown in Fig. 10, the current of the measurements obtained with the TCIR gave consistent results for both Eppendorf tubes. The average normalized current is 1.2002 ± 0.0013 pA/MBq, which is larger than the one measured for the sterile glass vials (1.0972 ± 0.0012 pA/MBq) by more than 8.5%. This shows the importance of the container used, as plastic attenuates the low-energy gamma rays less than glass.

Additional measurements were performed using other radionuclides to compare the response of the ionization chamber for Eppendorf tubes and sterile glass vials. Standardized samples of ^137^Cs, ^60^Co, ^133^Ba and ^57^Co were used to fill the two types of container with 1 g of solution. A chamber response difference of 0.23%, 0.21%, 0.42% and 0.60%, respectively, was determined (Table 3). The largest difference was observed for ^57^Co, which has two gamma emissions at 122.06 and 136.47 keV. It was concluded that for these four radionuclides, the same calibration factor could be used for Eppendorf tubes as for glass vials. It also showed that the attenuation of gamma rays above 100 keV could be neglected for these two vial types, assuming a fluctuation of lower than 1%.

**Table 3:** Response difference comparison of the ionization chamber measurements of ^161^Tb, ^137^Cs, ^60^Co, ^133^Ba and ^57^Co solutions in Eppendorf tubes and sterile glass vials

Radionuclides	Energy of gamma lines (keV)	Response difference (%)
^161^Tb	49–75	8.5
^137^Cs	662	0.23
^60^Co	1173–1332	0.21
^133^Ba	276–384	0.42
^57^Co	122–136	0.6

#### ^161^Tb activity measurement in GMP vial

**Fig. 11:**
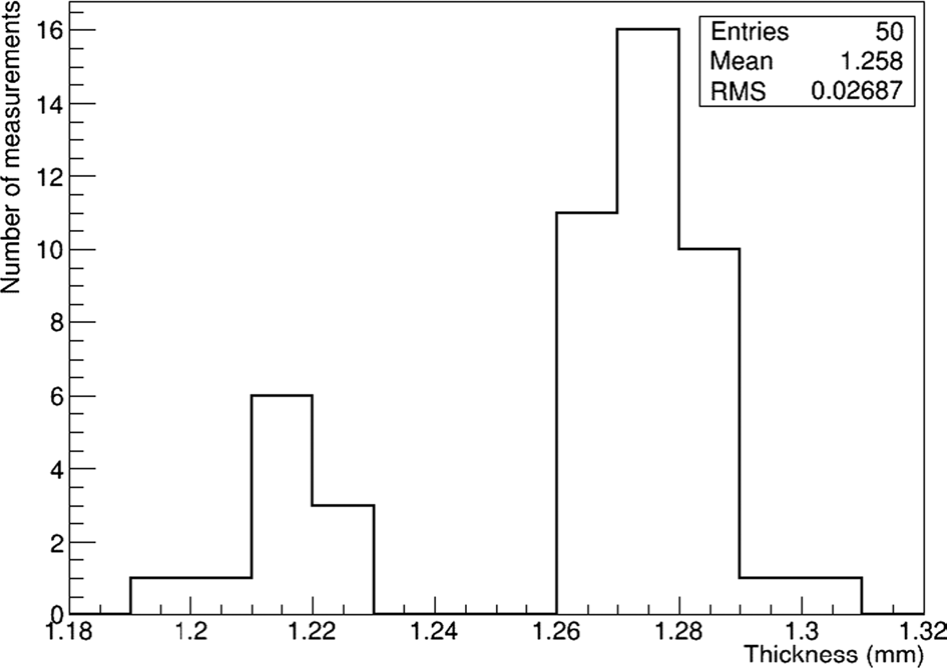
Distribution of the thickness measured for 10 points over the circumference of five GMP vials

The wall thickness of five GMP vials was measured with 10 points over its circumference. The results showed very good regularity, similar to the sterile glass vials. The RMS of the distribution was 27 μm (Fig. 11) and the thickness 1.26 mm, much thinner than the penicillin vial. The maximum fluctuation of the thickness for the same vial was 90 μm.

**Fig. 12:**
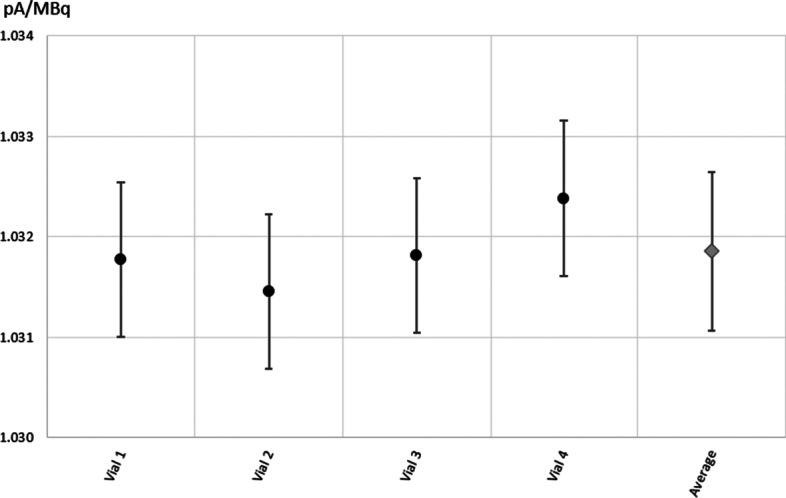
Normalized current values for the four GMP vials filled with 20 mL ^161^TbCl_3_ solution

Figure 12 gives the normalized current measured for the four GMP vials filled with ^161^Tb solution. The average value was 1.0319 ± 0.0008 pA/MBq, which was 6% and 14% smaller than for the sterile glass vial and Eppendorf tube, respectively. The difference between the two glass vials can be explained by the filling volume, which was 20 mL compared with 1 mL for the sterile glass vial and, therefore, the position of the radioactive solution inside the well was more distributed along the vertical axis, which changed the response of the chamber slightly.

**Fig. 13:**
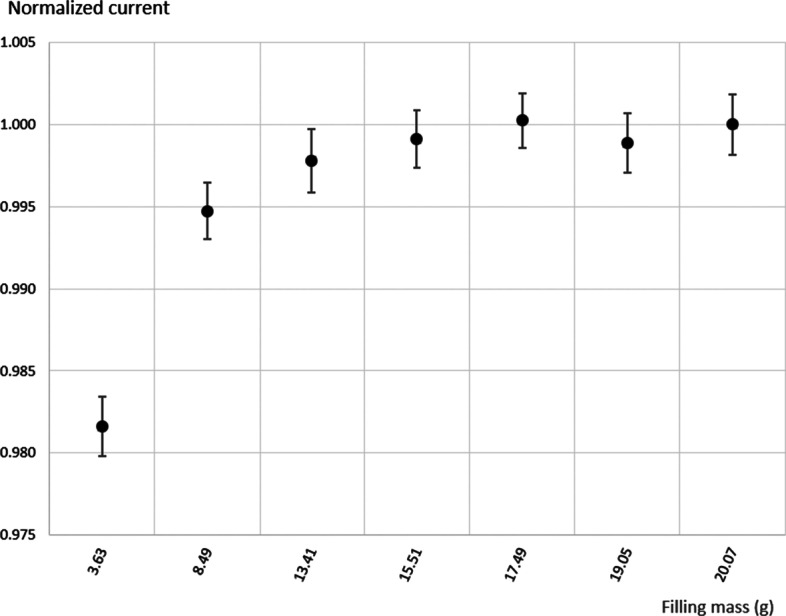
Normalized current values for different filling levels using a GMP vial

In order to compare the effect of the filling level, a GMP vial was filled with 3.6 g of the ^161^Tb stock solution and gradually made up to 20 g using nonradioactive (“cold”) Tb solution. After each addition, the vial was shaken to ensure a homogenous solution, weighed and, finally, centrifuged. The vial was measured at the TCIR and the measured currents were normalized to the value obtained with 20 g filling level. As shown in Fig. 13, the measured current started stabilizing around 14 g, however, the difference between filling at 8.49 g and 20 g was around 0.5%. From 5 g and below, a significant decrease, larger than 1%, of the current was measured.

## Discussion

The results obtained in this work demonstrate the importance of the solution volume, vial type, as well as the vial quality to ensure reproducible and accurate measurement of low-energy gamma-emitting radionuclides. Although some vial types can be suitable for measuring radionuclides with gamma-energy emissions above 100 keV, they are not necessarily suitable for the measurement of lower-energy gamma emissions.

The main gamma-emission lines of ^161^Tb are below 75 keV (Table 1). A difference of 4.5% was observed between two penicillin vials of the same type using the same ^161^Tb solution. Vials with regular thicknesses are, therefore, needed for the measurement of low-energy gamma emitters. Measurements using sterile and GMP glass vials, which have good thickness regularity, demonstrated their ability to measure low-energy gamma emitters more accurately. However, the response of the ionization chamber showed a significant difference between the two vial types of around 6%. This necessitates the use of different calibration factors for each type of vial. In addition, measurements with plastic Eppendorf tubes gave a response 8.5% higher than for sterile glass vials and 14% higher than that for GMP vials when measuring ^161^Tb. However, for radionuclides with gamma energies above 100 keV, the response differences were similar (Table 3). This shows that gamma attenuation has to be taken into account below 100 keV and, consequently, it is necessary to have different calibration factors for different containers used for production, preclinical studies and GMP production, respectively (Fig. 3). The measurement results for GMP vials using different ^161^Tb solution volumes gave no difference if the vial was filled at 10 g or more. The chamber response slightly decreased, by approximately 2%, for low filling levels around 3 g. As a result, the calibration factor would have to be corrected according to the volumes used in practice. This results are in agreement with studies using Monte Carlo simulation [3, 4], which could be an alternative for calculation of calibration factors. However it would require a precise knowledge of the geometry of the dose calibrator (size of chamber, thickness and material of the inner walls…) as well as for the used container geometry.

These results demonstrate the importance of the container type for the measurement of ^161^Tb and more generally for low-energy gamma emitters with ionization chambers and radionuclide dose calibrators. It is necessary to have a dedicated calibration factor for each container type to account for geometry, material and filling level in order to achieve an accurate activity assessment for low-energy gamma emitters (< 100 keV).

## Conclusion

The activity measurement of standardized ^161^Tb solution was performed using four different container types in an ionization chamber, showing significant discrepancies between vials that have insufficiently regular, or similar, geometry. A more regular vial type was chosen and standardized activity samples of ^161^Tb were measured, showing a stable response with the ionization chamber. It is important to highlight that, for glass vials, it would also be important to use even-shaped vials according to a specific standard in order to ensure consistent quality between the different production batches. Additionally, it was shown that Eppendorf tubes and glass vial containers have a similar response, within 1%, for gamma emitters above 100 keV, but their response can differ by 8.5% or 14% for ^161^Tb, which has 99% of its gamma emissions below 100 keV.

It is, therefore, very important to use the appropriate calibration factor according to the container, and also to take different filling volumes into account. Recalculating a calibration factor for each geometry condition (radionuclide, vial or syringe type, filling volume, etc.) is recommended for low-energy gamma emitters below 100 keV. For such radionuclides, using the wrong factor can lead to an assessment error of several percent of the activity in question. Ideally, in each practical case (radionuclide production, preclinical studies and radiopharmaceutical production), a calibration factor should be recalculated with a well-known source, traceable to a reference standard. It is worth mentioning that the results reported in this study will have particular importance for the precise activity measurement of the therapeutic radionuclide ^161^Tb for its clinical use.

## Data Availability

The data that support the findings of this study are available from the corresponding author upon reasonable request and with permission of the institution where measurement data was acquired.

## Abbreviations

PET: Positron emission tomography
SPECT: Single-photon emission computed tomography
RDC: Radionuclide dose calibrator
NMIs: Nuclear Metrology Institutes
CGPM: General Conference on Weights and Measures
BIPM: The Bureau of Weights and Measures
CIPM MRA: International Committee for Weights and Measures Mutual Recognition Agreement
CMCs: Calibration and Measurement Capabilities
SIR: Système International de Référence
KCDB: Key Comparison Data Base
CIR: Chamber d’ionization de référence
IRA: Institute of Radiation Physics
METAS: Swiss Federal Office of Metrology
IAEA: International Atomic Energy Agency
PSI: Paul Scherrer Institute
HPGe: High Purity Germanium
TDCR: Triple to Double Coincidence Ration method
RMS: Root Mean square
